# Sotatercept Rescue Therapy for Acute Right Heart Failure in HIV‐Associated PAH

**DOI:** 10.1002/pul2.70379

**Published:** 2026-07-27

**Authors:** Yaosi Li, Daniel Grund, Nils Kremer, Holger Müller‐Redetzky, Marie Zajonz, Christian Gaebler, Martin Witzenrath, Horst Olschewski, Athiththan Yogeswaran

**Affiliations:** ^1^ Department of Infectious Diseases Respiratory Medicine and Critical Care, Charité‐Universitätsmedizin Berlin Berlin Germany; ^2^ Department of Internal Medicine Universities of Giessen and Marburg Lung Center (UGMLC), Member of the German Center for Lung Research (DZL), Justus‐Liebig‐University Giessen Giessen Hesse Germany; ^3^ Institute for Lung Health (ILH), Cardio‐Pulmonary Institute (CPI) Giessen Hesse Germany

**Keywords:** HIV, intensive care medicine, pulmonary hypertension, right heart failure, sotatercept

## Abstract

Despite major advances in antiretroviral treatment, pulmonary arterial hypertension (PAH) remains a common, severe complication in patients diagnosed with HIV. Treatment of HIV‐associated PAH targets the nitric oxide, endothelin, and prostacyclin pathways, with limited evidence for novel therapies, as these patients have been excluded from most pivotal randomized controlled trials. We report a 58‐year‐old male with HIV‐PAH on dual oral PAH‐targeted therapy who developed acute right ventricular (RV) failure triggered by sepsis. Despite escalation of guideline‐directed therapy with intravenous prostacyclin and catecholamines, the patient continued to exhibit decompensated right heart failure. Sotatercept was initiated, after which the patient showed marked improvement within 24 h, including improved echocardiographic RV function, a decline in NT‐proBNP, recovery of renal function, and successful weaning from catecholamines and prostacyclin. The patient was subsequently transferred to a general ward in stable condition. This case suggests a potential role for sotatercept as rescue therapy in acute decompensated HIV‐PAH, with a rapid temporal association with hemodynamic improvement. Further randomized controlled studies are warranted in this high‐risk population.

## Take Home Message

Sotatercept may represent a potential rescue therapy in acute decompensated HIV‐associated PAH, with rapid improvement of clinical and right heart function despite insufficient response to standard therapy.

## 1 Introduction

Human immunodeficiency virus (HIV) infection is an established risk factor for developing pulmonary arterial hypertension (PAH) [1]. Approximately 0.5% of people living with HIV are diagnosed with PAH (HIV‐PAH), representing more than a 1000‐fold increased risk compared to the general population [1, 2]. While prior to the availability of antiretroviral therapy (ART) and PAH‐specific therapies, 3‐year survival of HIV‐PAH was below 50%, recent advances with contemporary treatment strategies combining ART and PAH‐targeted therapies have improved survival [1, 3].

Current treatment of HIV‐associated PAH largely follows that of idiopathic PAH, targeting the nitric oxide, endothelin, and prostacyclin pathways [1]. Although HIV‐PAH shares a multifactorial pathobiology that is partially comparable to other forms of Group 1 PH, its clinical management differs due to the exclusion of these patients from most pivotal randomized controlled trials [1, 4, 5, 6, 7]. Sotatercept, an activin signaling inhibitor, represents a novel therapeutic approach that rebalances the transforming growth factor‐beta (TGF‐β) superfamily pathway [4]. In the STELLAR trial, sotatercept added to background therapy, significantly improved exercise capacity, pulmonary hemodynamics, and NT‐proBNP levels in PAH patients [4]. Notably, the risk of death or clinical worsening was reduced by 84% compared with placebo [4]. Subsequent trials (ZENITH and HYPERION) confirmed the beneficial effects of sotatercept in high‐risk patients and those with early disease [8, 9].

Importantly, patients with HIV‐PAH were excluded from all sotatercept trials, resulting in a significant evidence gap regarding its efficacy in this population.

## 2 Case Report

A 58‐year‐old man living with HIV (initial diagnosis in 1992) on antiretroviral therapy since 1997, which was switched to dolutegravir, abacavir, and lamivudine in 2014, was diagnosed with PAH in 2024. He received combination PAH therapy with tadalafil and macitentan. Under this regimen, RV function remained preserved, with stable hemodynamics and no signs of right heart failure until recently.

In March 2026, the patient developed sepsis secondary to a severe urinary tract infection and was admitted to the intensive care unit (ICU, day 0). Despite prompt initiation of antimicrobial therapy, his clinical condition deteriorated rapidly, progressing to acute right heart failure.

At ICU admission, inflammatory markers were markedly elevated (C‐reactive protein (CRP) 141 mg/L, procalcitonin 98 µg/L). Empiric antimicrobial therapy with piperacillin/tazobactam was initiated and escalated to meropenem the following day due to persistent clinical instability. Vasopressor support with norepinephrine was started at admission, and vasopressin was added on day 1 due to ongoing hemodynamic instability.

Transthoracic echocardiography (TTE) demonstrated severe RV dysfunction, with a tricuspid annular plane systolic excursion (TAPSE) of 18 mm and TAPSE/PASP ratio of 0.35 mm/mmHg. The patient presented with dyspnea at rest (WHO functional class IV), peripheral edema, pleural effusions, and signs of combined backward and forward RV failure. Laboratory testing revealed markedly elevated NT‐proBNP, increased bilirubin, and impaired renal function (Table 1).

**TABLE 1:** Clinical course and response to sotatercept in HIV‐associated pulmonary arterial hypertension.

Parameter	ICU admission (Day 0)	Pre‐sotatercept (Day 3)	24 h post‐sotatercept (Day 4)	ICU discharge
Clinical parameters				
WHO functional class	IV	IV	III	II
Laboratory parameters				
NT‐proBNP, pg/mL	—	32,326	—	7027
eGFR, mL/min/1.73m^2^	< 15	34	96	58
GGT, U/L	92	98	—	87
Bilirubin, mg/dL	4,04	2,64	—	1,05
CRP, mg/L	141	53	30 (Day 5)	—
Echocardiography				
TAPSE, mm	18	11	18	30
TR gradient, mmHg	62	40	49	54
RV S′, cm/s	—	5	—	10
CVP, mmHg	—	—	—	4
TAPSE/PASP, mm/mmHg	0,29	0,27	0,37	0,56
PH‐specific therapy				
Iloprost, intravenous, ng/kg/min	—	2	2	0
Enoximon, intravenous, µg/kg/min	—	2	0	0
Levosimendan	—	12,5 mg/24 h	—	—
Catecholamines				
Norepinephrine, µg/kg/min	—	0,5	0,12	0
Vasopressin, IU/h	—	2,5	1,5	0

*Note:* The table summarizes key clinical, laboratory, and echocardiographic parameters across the disease course.

Therapy was intensified with intravenous iloprost (initiated on day 1 and rapidly uptitrated to 2 ng/kg/min) and an intravenous phosphodiesterase III inhibitor, which was later discontinued after initial stabilization. Levosimendan was added on day 2. Despite escalation of therapy, the patient remained critically ill, with further deterioration of RV dysfunction (TAPSE 11 mm, RV S′ 5 cm/s, TAPSE/PASP 0.27 mm/mmHg), persistent hemodynamic instability requiring high‐dose vasopressor support (up to 0.5 µg/kg/min norepinephrine and 2.5 IU/h vasopressin). Additionally, the patient was receiving high‐flow nasal oxygen therapy at a flow rate of 50 L/min and an FiO_2_ of 100%, with a PaO_2_/FiO_2_ ratio of 0.65.

Given the refractory, life‐threatening right heart failure despite improving infection parameters, rescue therapy with 0.7 mg/kg sotatercept was initiated on day 3 under ongoing vasopressor support (norepinephrine 0.5 µg/kg/min, vasopressin 2.5 IU/h) and intravenous iloprost (2.5 ng/kg/min).

Within 24 h after sotatercept administration, a marked improvement in RV function was observed, including an increase in TAPSE (18 mm) and TAPSE/PASP ratio (0.37 mm/mmHg). This was accompanied by a rapid reduction in vasopressor requirements (norepinephrine 0.07 µg/kg/min and vasopressin 1.5 IU/h on day 4), followed by complete discontinuation of norepinephrine on day 5 and vasopressin on day 6. Intravenous iloprost was discontinued on day 7. Echocardiographic recovery was paralleled by impressive clinical improvement, including resolution of dyspnea, regression of peripheral edema, and normalization of end‐organ function (Table 1).

After identification of *E. coli* and availability of susceptibility results, antibiotic therapy was de‐escalated to cefotaxime, which was accompanied by a steady decline in inflammatory markers (CRP 163 mg/L on day 1, CRP 30 mg/L on day 5). Antimicrobial therapy was discontinued on day 8 after sustained clinical improvement. Following successful discontinuation of intravenous therapies and cumulative negative fluid balance of 5 L, the patient was transferred from the intensive care unit to a general ward on low‐flow oxygen therapy.

## 3 Discussion

This case highlights the potential role of sotatercept as a rescue therapy in acute right heart failure due to HIV‐associated PAH, a population which has been systematically excluded from randomized controlled trials.

The pathobiology of HIV‐associated PAH is characterized, among other features, by dysregulated growth factor signaling, including the TGF‐β superfamily [10, 11]. HIV viral proteins, such as glycoprotein‐120, promote endothelial injury and oxidative stress, while cytokines, including interleukin‐6 and tumor necrosis factor‐α contribute to a proinflammatory and proliferative vascular phenotype [11]. Interestingly, HIV‐associated PAH does not appear to be directly related to the degree of immunodeficiency or viral suppression status [6]. Sotatercept is thought to restore these imbalances between pro‐ and anti‐proliferative signaling by acting as a ligand trap for activins and some other ligands of TGF beta receptors [4].

The rapid improvement, characterized by normalization of RV function and clinical recovery, was notable. While clinical trials demonstrated benefits over weeks to months, recent data suggest an early reduction in right ventricular afterload [12]. These findings support both structural remodeling and early functional vascular effects.

Regarding prostacyclin therapy, prospective data on de‐escalation in the context of sotatercept are scarce [4]. In PULSAR and STELLAR, background therapies, including prostacyclins remained unchanged. Although observational data suggest that sotatercept start is associated with a reduction in i.v. prostacyclin dose, the rapid discontinuation observed in this acutely decompensated patient extends beyond current evidence.

This observation has several limitations. Sotatercept was administered in the context of intensive care, including levosimendan, enoximone, intravenous iloprost, vasopressor support, and decongestion resulting in a cumulative negative fluid balance. Each of these interventions may independently improve right ventricular function and right ventricular–pulmonary arterial coupling. However, none of these measures improved the desperate hemodynamic situation. Furthermore, the patient was treated for severe urosepsis, a condition that may improve quite rapidly. Therefore, the observed temporal association between sotatercept administration and clinical improvement does not establish causality and should be regarded as hypothesis‐generating.

Taken together, this case highlights a potential role of sotatercept as a rescue therapy in severe decompensated HIV‐PAH. The relevance of sotatercept in HIV‐PAH patients requiring intensive care therefore warrants further investigation.

## Author Contributions

Conceptualization: Athiththan Yogeswaran and Yaosi Li. Clinical data collection and investigation: Athiththan Yogeswaran, Daniel Grund, and Yaosi Li. Data analysis and interpretation: Athiththan Yogeswaran and Yaosi Li. Manuscript drafting: Athiththan Yogeswaran. Critical revision and editing: all authors. All authors approved the final version of the manuscript.

## Ethics Statement

Ethics approval was not required for this case report. Written informed consent for publication of the case details was obtained from the patient.

## Conflicts of Interest

Yaosi Li and Daniel Grund report no conflicts of interest. Nils Kremer has received speaker and consultancy fees from Janssen, AOP, OrphaCare, and MSD outside the submitted work. Holger Müller‐Redetzky and Marie Zajonz report no conflicts of interest. Christian Gaebler received travel support from Gilead Sciences for participating at scientific meetings. Martin Witzenrath has received research funding from Aptarion, Pantherna, and Biotest outside the current study, and lecture and advisory fees from AstraZeneca, Chiesi, Insmed, Gilead, Pfizer, Boehringer Ingelheim, Biotest, Pantherna, and Aptarion. Horst Olschewski has received lecture fees from AOP, Ferrer, and MedUpdate Europe; served as an adjudication board/Data Safety Monitoring Board member for Aerovate, Astra Zeneca, Bayer, IQVIA, Liquidia, Inhibikase, and Pulmovant; and acted as consultant for Paul Weiss, Menarini, MSD, and Liquidia. Athiththan Yogeswaran reports personal fees from MSD and Ferrer, and other support from AOP and OrphaCare.

## Policy on Using ChatGPT and Similar AI Tools

ChatGPT (OpenAI) was used solely for language editing and improvement of manuscript readability. No AI tools were used for scientific content generation, data analysis, interpretation, or conclusions. All scientific content was created and verified by the authors.

## Data Availability

The data that support the findings of this study are available from the corresponding author upon reasonable request.
